# Editorial: Nutrition and headaches: from long-held beliefs to scientific evidence

**DOI:** 10.3389/fnut.2023.1353427

**Published:** 2024-01-08

**Authors:** Laís Bhering Martins, Arman Arab, Antonio Lucio Teixeira, Adaliene Versiani Matos Ferreira

**Affiliations:** ^1^Departamento de Nutrição, Escola de Enfermagem, Universidade Federal de Minas Gerais, Belo Horizonte, Minas Gerais, Brazil; ^2^Department of Psychiatry and Behavioral Sciences, University of Texas Health Science Center, Houston, TX, United States; ^3^Division of Sleep Medicine, Harvard Medical School, Boston, MA, United States; ^4^Medical Chronobiology Program, Division of Sleep and Circadian Disorders, Department of Medicine and Neurology, Brigham and Women's Hospital, Boston, MA, United States

**Keywords:** migraine, dietary diversity score, ketogenic diet, potassium, resting energy expenditure

Migraine is a common primary headache with increasing prevalence that results in significant functional limitations. It is currently ranked as the second leading global cause of disability (1, 2).

Despite its high prevalence and impact, the underlying mechanisms of migraine are still not well understood. Nutrition-related factors, such as the composition of one's diet and its quality, have been identified as potential triggers for migraine attacks or as contributing risk factors for the development of migraine (3). However, a recent systematic review has highlighted the need for high-quality studies to establish the diet-migraine association (4).

In light of the fact that a significant number of individuals with headache disorders discontinue pharmacological treatments due to the limited efficacy and side effects (5), exploring alternative and complementary strategies becomes crucial for improving patients' quality of life.

Therefore, this Research Topic aims to showcase studies on the association between nutrition-related factors and migraine and the putative mechanisms underlying this link. It includes a Research Topic of four research articles, including one meta-analysis.

Two studies in this Research Topic support the hypothesis that diet composition can contribute to enhanced management of migraine headaches. In a meta-analysis of randomized controlled trials and uncontrolled observational studies, Neri et al. observed an overall positive effect of ketogenic interventions, including a very-low-calorie ketogenic diet, a modified Atkins diet, a classic ketogenic diet, and the administration of an exogenous source of beta-hydroxybutyrate on reducing the frequency and severity of migraine episodes. However, due to the heterogeneity of the studies, the authors reinforce the need for more investigations and recommend the use of measuring ketone levels during ketogenic therapy to monitor treatment adherence.

Additionally, in a cross-sectional study, Amani Tirani et al. demonstrate a significant inverse association between the dietary diversity score (DDS) and the frequency of migraine headaches. The DDS is a metric calculated based on food group consumption that can indicate diet quality and micronutrient adequacy (6). While DDS was related to attack frequency, it showed no association with severity or migraine-related disability. Their observations also highlight a negative relationship between nitric oxide (NO) levels and DDS. Indeed, NO may play a role in migraine pathophysiology, and earlier studies have suggested the possibility of using NO synthase (NOS) inhibitors as a therapeutic strategy to alleviate migraines (7). NO plays a pivotal role in maintaining the vascular tone among its biological functions. Potassium also regulates cell membrane potential and plays a role in vascular tone. In this context, the study by Xu et al. explores the relationship between potassium intake and self-reported severe headaches or migraines. Xu et al. used data from the National Health and Nutrition Examination Survey (NHANES) spanning 1999–2004 and found a non-linear L-shaped correlation between dietary potassium intake and reported severe headaches or migraines. They identified an inflection point at 1439.3 mg/day. According to their analyses, each 0.1 g increase in daily potassium intake was associated with a 4.8% decrease in the likelihood of reporting severe headaches or migraines up to the threshold of 1439.3 mg/day. Increasing potassium intake did not appear to provide additional benefits in preventing migraines beyond this threshold.

In a cross-sectional study, Martins et al. show that women with migraines exhibit higher resting energy expenditure (REE) than those without. This observation highlights the importance of accurately assessing biological characteristics for tailored nutritional interventions for these patients and suggests the association between pain and energy expenditure. REE is an important measure for determining more precise nutritional interventions and can also be calculated using predictive formulas. Thus, this study further assesses which predictive formulas would be most accurate for evaluating the REE of women with migraine. The Mifflin-St Jeor and Henry and Rees formulas were found to be the most accurate predictive formulas for estimating REE in women experiencing migraines.

In conclusion, these studies shed light on the potential impact of nutrition in managing migraines, presenting opportunities for personalized interventions based on individual biological profiles.

## Author contributions

LBM: Writing—original draft, Writing—review & editing. AA: Writing—review & editing. ALT: Writing—review & editing. AVMF: Writing—review & editing.
